# Supplementary material for: Effectiveness of virtual laboratory in engineering education: A meta-analysis
Source: PLoS One. 2024 Dec 30;19(12):e0316269. doi: 10.1371/journal.pone.0316269 (PMC11684589; doi:10.1371/journal.pone.0316269)
Supplement: S3 Appendix — (DOCX) [file pone.0316269.s003.docx]

| **Model** | **Group by Subgroup** | **Study name** | **Subgroup within study** | **Statistics for each study** | | | | | | | | **Weight (Separate tau) Relative weight** |  |
| --- | --- | --- | --- | --- | --- | --- | --- | --- | --- | --- | --- | --- | --- |
|  |  |  |  | **Hedges's g** | \| **Standard error** \| **Variance** \| **Lower limit** \| **Upper limit** \| **Z-Value** \| \| --- \| --- \| --- \| --- \| --- \| | | | | | | **p-Value** |  |  |
| **Random** | **L** | **(Ogbuanya**  **(Wang et (Wang et (Tu li et al.,** | **L**  **A A A** | **3.571** | | **0.270** | **0.073** | **3.042** | **4.099** | **13.233** | **0.000** | **100.00** 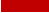  **8.82** 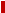 **8.80** 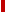 **7.90** 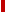 |  |
|  | **L** |  |  | **3.571** | | **0.270** | **0.073** | **3.042** | **4.099** | **13.233** | **0.000** |  |  |
|  | **A A A** |  |  | **0.274 0.564 1.543** | | **0.133 0.135 0.219** | **0.018 0.018 0.048** | **0.012 0.299 1.114** | **0.535 0.830 1.972** | **2.051 4.168 7.044** | **0.040 0.000 0.000** |  |  |
|  | **A A A A A A A** | **(Lei et al., (Lei et al., (Gamo,**  **(Ogbuanya (Lei et al., (Lei et al., (Lei et al.,** | **A A A A A A A** | **0.112 0.252 0.017 2.573 0.254 0.098 0.406** | | **0.091 0.102 0.245 0.226 0.102 0.105 0.166** | **0.008 0.010 0.060 0.051 0.010 0.011 0.027** | **-0.066 0.052 -0.463 2.129 0.054 -0.107 0.081** | **0.291 0.453 0.496 3.016 0.455 0.303 0.731** | **1.231 2.467 0.068 11.368 2.487 0.941 2.450** | **0.218 0.014 0.946 0.000 0.013 0.347 0.014** | **9.15** 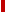 **9.07** 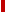 **7.59** 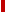 **7.81** 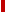 **9.07** 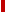 **9.06** 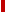 **8.50** 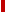 |  |
| **Random** | **A A** | **(Chang et (Amirkhani**  **(Prasetya et** | **A A**  **C** | **0.300 0.861** | | **0.194 0.372** | **0.038 0.139** | **-0.080 0.132** | **0.681 1.591** | **1.546 2.314** | **0.122 0.021** | **8.19** 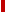 **6.02** I  **16.79** 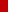 |  |
|  | **A** |  |  | **0.574** | | **0.151** | **0.023** | **0.277** | **0.871** | **3.791** | **0.000** |  |  |
|  | **C** |  |  | **2.804** | | **0.355** | **0.126** | **2.108** | **3.501** | **7.890** | **0.000** |  |  |
|  | **C C C C** | **(Prasetya et (Sapriadil et (Wiesner**  **(Wiesner** | **C C C C** | **2.184 1.329 -0.683 -1.263** | | **0.318 0.262 0.430 0.456** | **0.101 0.068 0.185 0.208** | **1.560 0.816 -1.525 -2.157** | **2.808 1.842 0.159 -0.369** | **6.859 5.077 -1.591 -2.768** | **0.000 0.000 0.112 0.006** | **16.96** 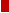 **17.19** 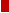 **16.41** 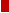 **16.26** 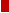 |  |
| **Random** | **C** | **(Wiesner** | **C** | **-0.687** | | **0.430** | **0.185** | **-1.529** | **0.156** | **-1.598** | **0.110** | **16.41** 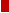 |  |
|  | **C** |  |  | **0.639** | | **0.648** | **0.420** | **-0.630** | **1.909** | **0.987** | **0.324** |  |  |
|  | **CL** | **(Dong et al.,** | **CL** | **-5.213** | | **0.559** | **0.312** | **-6.307** | **-4.118** | **-9.331** | **0.000** | **31.64** 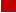 |  |
| **Random** | **CL CL** | **(Singh et (Singh et**  **(Ogbuanya** | **CL CL**  **E** | **-0.761 -0.579** | | **0.256 0.260** | **0.066 0.068** | **-1.262 -1.090** | **-0.259 -0.069** | **-2.972 -2.226** | **0.003 0.026** | **34.19** 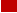 **34.17** 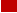  **100.00** 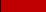 |  |
|  | **CL** |  |  | **-2.107** | | **1.023** | **1.046** | **-4.112** | **-0.103** | **-2.060** | **0.039** |  |  |
|  | **E** |  |  | **2.888** | | **0.239** | **0.057** | **2.419** | **3.357** | **12.066** | **0.000** |  |  |
| **Random** | **E** | **(?lvarez**-**Mar** | **K** | **2.888** | | **0.239** | **0.057** | **2.419** | **3.357** | **12.066** | **0.000** | **7.18** 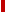 |  |
|  | **K** |  |  | **1.920** | | **0.461** | **0.212** | **1.017** | **2.824** | **4.166** | **0.000** |  |  |
|  | **K K K K K K K K K K** | **(Dong et al., (Srinivasa et (Singh et**  **(Rasheed et (Ramos et (Koll?ffel**  **(Shyr, 2010) (Wiesner**  **(Wiesner (Wiesner** | **K K K K K K K K K K** | **7.841 0.698 1.087 0.557 0.303 0.960 0.632 0.014 -0.270 0.322** | | **0.786 0.189 0.265 0.150 0.270 0.317 0.344 0.418 0.420 0.421** | **0.618 0.036 0.070 0.022 0.073 0.101 0.118 0.175 0.176 0.177** | **6.300 0.329 0.568 0.264 -0.226 0.337 -0.041 -0.805 -1.092 -0.503** | **9.383 1.068 1.606 0.851 0.832 1.582 1.305 0.834 0.553 1.146** | **9.971 3.702 4.102 3.723 1.124 3.023 1.840 0.034 -0.642 0.765** | **0.000 0.000 0.000 0.000 0.261 0.003 0.066 0.973 0.521 0.444** | **5.09** 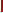 **8.75** 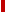 **8.39** 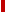 **8.89** 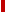 **8.37** 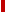 **8.10** 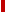 **7.94** 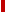 **7.47** 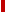 **7.46** 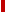 **7.45** 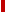 |  |

| **Model** | **Group by Subgroup** | **Study name** | **Subgroup within study** | **Statistics for each study** | | | | | | | **Weight (Separate tau) Relative weight** |  |
| --- | --- | --- | --- | --- | --- | --- | --- | --- | --- | --- | --- | --- |
|  |  |  |  | **Hedges's g** | **Standard error** | **Variance** | **Lower limit** | **Upper limit** | **Z-Value** | **p-Value** |  |  |
|  | **K** | **(Wiesner** | **K** | **-0.433** | **0.423** | **0.179** | **-1.261** | **0.396** | **-1.024** | **0.306** | **7.44** 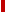 |  |
| **Random** | **K** | **(Wiesner**  **(Dong et al., (Singh et** | **K**  **L L** | **0.000** | **0.418** | **0.175** | **-0.819** | **0.819** | **0.000** | **1.000** | **7.47** 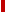  **32.08** 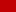 **33.94** 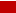 |  |
|  | **K** |  |  | **0.865** | **0.267** | **0.071** | **0.343** | **1.388** | **3.245** | **0.001** |  |  |
|  | **L L** |  |  | **2.874 0.901** | **0.378 0.259** | **0.143 0.067** | **2.132 0.393** | **3.615 1.410** | **7.594 3.473** | **0.000 0.001** |  |  |
|  | **L** | **(Singh et** | **L** | **0.340** | **0.257** | **0.066** | **-0.163** | **0.844** | **1.326** | **0.185** | **33.98** 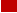 |  |
| **Random** | **L** | **(Singh and** | **O** | **1.343** | **0.667** | **0.444** | **0.037** | **2.650** | **2.015** | **0.044** | **15.12** 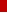 |  |
|  | **O** |  |  | **1.708** | **0.259** | **0.067** | **1.200** | **2.217** | **6.586** | **0.000** |  |  |
|  | **O O O O** | **(Singh et (Chang et (Chang et (Wiesner** | **O O O O** | **1.326 0.357 1.036 -0.572** | **0.282 0.137 0.143 0.426** | **0.080 0.019 0.020 0.182** | **0.773 0.090 0.755 -1.407** | **1.879 0.625 1.317 0.263** | **4.700 2.615 7.236 -1.342** | **0.000 0.009 0.000 0.180** | **14.73** 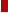 **16.88** 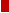 **16.81** 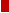 **12.13** 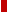 |  |
| **Random** | **O O** | **(Wiesner (Wiesner** | **O O** | **-0.495 0.506** | **0.424 0.424** | **0.180 0.180** | **-1.327 -0.325** | **0.336 1.338** | **-1.168 1.193** | **0.243 0.233** | **12.17** 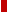 **12.16** 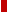 |  |
|  | **O** |  |  | **0.620** | **0.265** | **0.070** | **0.101** | **1.139** | **2.340** | **0.019** |  |  |
