# Supplementary material for: Effectiveness of virtual laboratory in engineering education: A meta-analysis
Source: PLoS One. 2024 Dec 30;19(12):e0316269. doi: 10.1371/journal.pone.0316269 (PMC11684589; doi:10.1371/journal.pone.0316269)
Supplement: Attachment — Submitted filename: Response to reviewers.docx [file pone.0316269.s004.docx]

Note: To ensure the accuracy of language use, spelling, and grammar, we utilized the scientific editing services recommended by the journal, Editage, and have explicitly acknowledged this in the acknowledgments section. To distinguish the changes made to the content in this revision, the "Revised Manuscript with Track Changes" document highlights in yellow the modifications made by the authors in response to the reviewers' comments, and highlights in cyan the changes made to the language.

Responds to each point raised by the academic editor：

1. Please ensure that your manuscript meets PLOS ONE's style requirements, including those for file naming.

**Respond:** All figures, tables, and appendix have been named as required.

2. Thank you for stating the following financial disclosure:

“Major science and technology projects of Autonomous Region:

Integration and demonstration of key technologies for improving quality, increasing yield and processing comprehensive utilization of walnut

Item No. : (2021A02002-2)”

Please state what role the funders took in the study. If the funders had no role, please state: "The funders had no role in study design, data collection and analysis, decision to publish, or preparation of the manuscript."

If this statement is not correct you must amend it as needed.

Please include this amended Role of Funder statement in your cover letter; we will change the online submission form on your behalf.

**Respond**: It has been stated as requested in the cover letter.

3. When completing the data availability statement of the submission form, you indicated that you will make your data available on acceptance. We strongly recommend all authors decide on a data sharing plan before acceptance, as the process can be lengthy and hold up publication timelines. Please note that, though access restrictions are acceptable now, your entire data will need to be made freely accessible if your manuscript is accepted for publication. This policy applies to all data except where public deposition would breach compliance with the protocol approved by your research ethics board. If you are unable to adhere to our open data policy, please kindly revise your statement to explain your reasoning and we will seek the editor's input on an exemption. Please be assured that, once you have provided your new statement, the assessment of your exemption will not hold up the peer review process.

**Respond:** Agree to the data sharing plan.

4. Please include a separate caption for each figure in your manuscript.

**Respond**: A separate title has been added to each figure.

5. As required by our policy on Data Availability, please ensure your manuscript or supplementary information includes the following:

A numbered table of all studies identified in the literature search, including those that were excluded from the analyses.

For every excluded study, the table should list the reason(s) for exclusion.

If any of the included studies are unpublished, include a link (URL) to the primary source or detailed information about how the content can be accessed.

**Respond**: It has been added in the attachment named “Appendix 2. Publications identified in the literature search”.

A table of all data extracted from the primary research sources for the systematic review and/or meta-analysis. The table must include the following information for each study:

Name of data extractors and date of data extraction

Confirmation that the study was eligible to be included in the review.

All data extracted from each study for the reported systematic review and/or meta-analysis that would be needed to replicate your analyses.

If data or supporting information were obtained from another source (e.g. correspondence with the author of the original research article), please provide the source of data and dates on which the data/information were obtained by your research group.

**Respond**: It has been added in the attachment named “Appendix 1. All data extracted from the publications that meet the inclusion criteria”.

If applicable for your analysis, a table showing the completed risk of bias and quality/certainty assessments for each study or outcome. Please ensure this is provided for each domain or parameter assessed. For example, if you used the Cochrane risk-of-bias tool for randomized trials, provide answers to each of the signalling questions for each study. If you used GRADE to assess certainty of evidence, provide judgements about each of the quality of evidence factor. This should be provided for each outcome.

**Respond**: It has been added in the attachment named “Appendix 1. All data extracted from the publications that meet the inclusion criteria”.

An explanation of how missing data were handled.

**Respond**: There is no missing data.

6. Please review your reference list to ensure that it is complete and correct. If you have cited papers that have been retracted, please include the rationale for doing so in the manuscript text, or remove these references and replace them with relevant current references. Any changes to the reference list should be mentioned in the rebuttal letter that accompanies your revised manuscript. If you need to cite a retracted article, indicate the article’s retracted status in the References list and also include a citation and full reference for the retraction notice.

**Respond**: No retracted papers were cited. Changes to the reference list have been highlighted.

Responds to each point raised by Reviewer #1：

1. The authors’ goal of summarizing findings into the types of outcomes (academic achievement, skills, etc.) is very interesting. It seems to me that individual experiments may be very different from each other even when addressing the same type of outcome. I assume in these studies there are difference in data type such as quantitative versus qualitative, differences in assessment methods, and differences in protocol. Is the method of Hedge’s g able to help make statements about the effectiveness when the underlying studies are different? If so, I think this argument could be made stronger.

**Respond**: Thanks for the valuable reviews. As you pointed out, even when addressing the same type of outcomes, there can be significant differences between individual experiments. Hedge's g is a common method used to measure effect size, especially when dealing with mean comparisons (such as differences between two groups). In this study, publications that are irrelevant to the theme and those based solely on students' subjective feedback (such as studies assessing the perceived usability and user satisfaction of various forms of virtual laboratories) have been excluded during the literature screening. Only studies that are relevant to the topic and based on objective assessment criteria (such as skill tests and examinations) and that employed a two-group research design were included. These efforts were made to ensure consistency in research outcomes and types of experiments. Additionally, the results from the sensitivity analysis in section Sensitivity analysis also indicated that the diversity of the studies did not affect the robustness of the results.

In response to the reviewers' comments, additional clarification has been provided in Section Abstract reading and full text sifting of the manuscript to ensure completeness and rigor in the discussion: publications that were irrelevant to the theme and those based solely on students’ subjective feedback (such as studies assessing the perceived usability and user satisfaction of various forms of virtual laboratories) were excluded. Ultimately, publications that aligned with the theme and were based on objective evaluation criteria (such as skill tests and exams) were included. These studies employed a two-group research design to compare the effects of various forms of virtual laboratories with traditional laboratories on engineering students. Additionally, these studies reported necessary descriptive summary statistics.

In addition, add the following explanation in section Sensitivity analysis: The overall effect size estimate (Hedges’ g) varies between approximately 0.590 and 0.789 when different studies are excluded, and the change magnitude is approximately 0.2. Thus, overall, the intervention has a positive and significant impact on the outcomes, and the change in effect size is relatively mild, with no significant fluctuations. That is to say, despite the methodological differences among various studies, the results demonstrate good robustness.

2. I did not understand the objective or reasoning for the analysis of moderator effect. Why where the categories of paper/author nation and year chosen? What was the intended goal of looking at these and what do they show? It seems that there is a fairly small number of studies from the different countries. Does that affect any meaning the reader is to understand from the moderator affect analysis results?

**Respond**: Thank you for raising this question. The reason for selecting country/region and publication year as variables for the moderating effect is to verify whether the context of different countries or regions influences the research results. Additionally, changes in the social environment over time and the continuous development of virtual experimental technologies may impact the research findings.

Reviewer’s suggestion is very insightful and highlights a flaw in my discussion. To ensure a more comprehensive discussion, additional clarification has been provided in Discussion section: Based on the analysis of moderator effect sizes, we have found that there are certain differences in effect sizes among different countries/regions and publication years. Publications from Nigeria show a significant effect size (2.995), while publications from the United States indicate an extremely low effect size (0.006). This suggests that there may be stronger factors or contextual conditions in Nigeria that make the relevant effects more pronounced, therefore, we speculate that the differences in effect sizes in our research perhaps due to the varying developments in social, economic, or cultural contexts across different countries. However, due to the limited number of studies from different countries, the analysis of the moderating effects in this research can only serve as a hypothesis and reference. We hope that future research will provide more reliable evidence on this issue.

Besides, the effect sizes based on publication year indicate that the recent study (2023) shows a higher effect size (2.340), while the study from 2004 displays a negative effect size (-0.311). This phenomenon often suggests that the latest research provides stronger support and evidence for the phenomenon of interest, which may be related to improvements in scientific research methods, enhanced data collection, and refined theories. In this meta-analysis, it is possible to infer that the technology of virtual laboratories has evolved over time, and there has been an increased attention to this research field. Although the analysis reveals the moderation effect of publication year, it remains important to consider the representativeness of the samples and the limitations of research design, such as potential selection bias and unequal sample sizes.

Some minor issues:

• Starting around the methods section, there are a few minor grammatical errors that don’t significantly affect the understanding but can be somewhat confusing or distracting.

• A few of the citations in the figures have formatting error question marks.

• In figure 4, the “L” classification is in two groups. Is there a reason for the separation? If so, that wasn’t conveyed in the paper.

**Respond**: To ensure the accuracy of language use, spelling, and grammar, we utilized the scientific editing services recommended by the journal, Editage, and have explicitly acknowledged this in the acknowledgments section. To distinguish the changes made to the content in this revision, the "Revised Manuscript with Track Changes" document highlights in yellow the modifications made by the authors in response to the reviewers' comments, and highlights in cyan the changes made to the language.

The question raised by the reviewer regarding the classification of "L" in Figure 4 into two groups is due to the involvement of multiple independent studies in some literature, necessitating a distinction in the forest plot. To address this issue, additional clarification has been included in Section Computation of summary effect sizes: It is worth noting that, due to the involvement of multiple independent studies in some publications, the following distinctions have been made in all the forest plots: a suffix of "A" indicates independent studies focused on students' Academic achievement; "O" indicates independent studies focused on students' Operational skill; "C" indicates independent studies on Cognitive ability; "K" indicates independent studies focused on students' Knowledge acquisition; "CL" indicates independent studies on students' Cognitive load; "L" indicates independent studies focused on students' Learning motivation; and "E" indicates independent studies focused on students' Learning Engagement. If the same publication involves multiple similar studies, add Arabic numerals after the uppercase letter suffix for differentiation.

Responds to each point raised by Reviewer #2:

The paper could be enhanced by following the following revisions and based on some of the revisions, you will need to demonstrate why this study is actually needed and has not been done in part before (and if so, take that into account and build the rationale for doing this study).

1) Use a framework (like PICO or other suitable) to frame your research questions, search terms, inclusion/exclusion criteria from the stated objectives as that will help in later organisation of the work. This will be particularly helpful in check list item 5 (how studies were grouped). Also for 10a and 10b in the checklist, this framework approach will help. you need to detail the same however.

**Respond:** I appreciate the reviewer’s constructive feedback; it has been very helpful to me. Based on the review comments, in section Define inclusion and exclusion, the application of the PICO framework is explained for constructing the research questions, search terms, and the inclusion/exclusion criteria derived from the established objectives of this study.

2) Need to specify how the quality of the work shortlisted was assessed and how the work of shortlisting and quality assessment was shared with the authors (state clearly who did what and how did you maintain reliability where more than one author did the work). Interrater stats needed as multiple authors are implied to have worked here.

**Respond:** Based on the review comments, in section Abstract reading and full text sifting, we describe the division of work, reliability maintenance, and feedback on results used in the literature inclusion/exclusion review.

3) For 13a in the checklist - Tabulate each study showing its chars. And comment on how they were eligible for grouping and synthesis. Group studies in a separate table showing how your grouped them for synthesis. Show the full table for 13c.

**Respond:** It has been added in the attachment named “Appendix 2. Publications identified in the literature search” and “Appendix 1. All data extracted from the publications that meet the inclusion criteria”. The "Appendix 1. All data extracted from the publications that meet the inclusion criteria" lists the intervention characteristics of each individual research, the types of outcomes used as subgroup classification criteria, the reasons for confirming that the individual study meets the screening criteria, and the risk of bias analysis.

4) what software was used to calculate statistics. There are standard software available, please use them to make it standard.

**Respond:** Based on the review comments, we have added a description of the literature management software EndNote X8 used to ensure the reliability of information filtering and statistical results in section Abstract reading and full text sifting. And we added the description that we used Comprehensive Meta Analysis software for data calculations and figure creation to ensure the reliability of the results in section Results.

5) for 13f, Describe this better than its been done. Process is stated, no evaluation of the values has been presented.

**Respond:** The evaluation of the values has been added in section Sensitivity analysis: Sensitivity analysis was performed to evaluate whether the pooled effect size was influenced by individual studies, that is, to assess the influence of the individual studies on the overall results by omitting one study at a time. For this sensitivity analysis, a summary of the effect sizes was compared together with their 95% CIs and the results confirmed the robustness to all (*n*=46) one-by-one study removals, the width of the confidence intervals varied slightly when different studies were excluded, but overall, they did not cross zero, indicating that the results are statistically significant. All analyzed Z values are greater than1.96, and the p values are less than 0.0001, indicating that regardless of which study is excluded, the results are statistically very significant (Fig 4). The overall effect size estimate (Hedges's g) varies between approximately 0.590 and 0.789 when different studies are excluded, the change magnitude is approximately 0.2. This indicates that, overall, the intervention has a positive and significant impact on the outcomes, the change in effect size is relatively mild, with no significant fluctuations. That is to say, despite the methodological differences among various studies, the results demonstrate good robustness.

6) for 17 and 18 on the checklist, you must provide this.

**Respond:** Provided in “Appendix 1. All data extracted from the publications that meet the inclusion criteria”.

7) The discussion section (23 in checklist) is weak and needs more work.

**Respond:** Additional discussion content has been added, including any limitations of the evidence included in the review and any limitations of the review process used.
